# Endourology (Lithiasis). Management, surgical considerations and follow-up of patients in the COVID-19 era

**DOI:** 10.1590/S1677-5538.IBJU.2020.S105

**Published:** 2020-07-27

**Authors:** Moisés E. Rodríguez Socarrás, Francesco Esperto, Marcelo Denilson Bapstistussi, Felipe Barufaldi, Matheus Soares Vital, Rocco Papalia, Annamaria Salerno, Rita Cataldo, Ana María Autrán-Gómez, Roberto Mario Scarpa

**Affiliations:** 1 Instituto de Cirugía Urólogica Avanzada Madrid Spain Instituto de Cirugía Urólogica Avanzada (ICUA), Madrid, Spain; 2 University of Rome Campus Biomedico Department of Urology Rome Italy Department of Urology, Campus Biomedico, University of Rome, Rome, Italy; 3 European Society of Residents in Urology Arnhem The Netherlands European Society of Residents in Urology (ESRU), Arnhem, The Netherlands; 4 Beneficência Portuguesa Hospital Department of Urology São PauloSP Brasil Department of Urology, Beneficência Portuguesa Hospital, São Paulo, SP, Brasil; 5 American Confederation of Urology Department of Lithiasis Buenos Aires Argentina Department of Lithiasis of American Confederation of Urology (CAU), Buenos Aires, Argentina; 6 University of Rome Unit of Anesthesia, Intensive care and pain management Department of Medicine Rome Italy Department of Medicine, Unit of Anesthesia, Intensive care and pain management, Campus Biomédico, University of Rome, Rome, Italy; 7 University Hospital Fundación Jiménez Díaz Department of Urology Madrid Spain Department of Urology, University Hospital Fundación Jiménez Díaz, Madrid, Spain

**Keywords:** COVID-19 [Supplementary Concept], spike protein, SARS-CoV-2 [Supplementary Concept], Lithotripsy

## Abstract

**Purpose:**

To provide recommendations on the endourological management of lithiasis in the scenario of the COVID-19 pandemic.

**Materials and Methods:**

A non-systematic review in PubMed and the grey literature, as well as recommendations by a panel of stakeholders was made, regarding management, surgical considerations and follow-up of patients affected by lithiasis in the COVID-19 era.

**Results:**

Under the current outbreak and COVID-19 pandemic scenario, patients affected by lithiasis should be prioritized into low, intermediate and high risk categories, to decide their delay and save resources, healthcare personnel, beds and ventilators. However, patients with potentially serious septic complications need emergency interventions. The possibility of performing or restarting elective activity depends on local conditions, the availability of beds and ventilators, and the implementation of screening protocols in the context of the COVID-19 pandemic. Delaying lithiasis surgery and increasing waiting lists will have consequences and will require considerable additional effort. Teleconsultation may be useful in guiding these patients, reducing visits and unnecessary exposure.

**Conclusions:**

categorization and prioritization of patients affected by lithiasis is crucial for management, surgical selection and follow-up. Protocols, measures and additional efforts should be carried out in the current situation of the COVID-19 pandemic.

## INTRODUCTION

The appearance of COVID-19 has dramatically influenced our lives, as well as medicine and urology practices. The epidemic began in December 2019 in China, to spread voraciously worldwide and become a pandemic early 2020.

The reality and policies are different among regions and countries. During the outbreak, all health efforts and resources are addressed to COVID-19 patients and to contain the contagion. Recommendations were made to prioritize surgeries, basically to postpone and reschedule non-urgent procedures to avoid unnecessary visits and spare resources, including health professionals, beds and ventilators( 1 - 3 ).

Lithiasis management includes a wide spectrum from asymptomatic patients to patients requiring a non-delayable intervention, (e.g. lumbar pain and fever due to obstructive lithiasis). There is a lack of consistent evidence regard lithiasis management in the current COVID-19 pandemic scenario. Safety and feasibility of elective lithiasis surgery is variable and uncertain, depending on local conditions as well as availability of resources. The impact on the activity of urology departments and clinical practice is clear and delay in treatment may bring consequences( 4 , 5 ). Moreover, the implementation of protocols to adapt to the “new normal” requires additional efforts.

We understand the heterogeneity and asymmetry of the pandemic among the countries under CAU umbrella. We only express the opinion and recommendations of a panel of stakeholders, based on the available literature and clinical experience in attempt to achieve the best possible practices on lithiasis management, in a highly changing scenario that may not represent the reality on all countries and regions; requiring adaptation with the best clinical sense, based on the evolution of evidence and local conditions.

We aim to provide recommendations on the endourological management of lithiasis in the scenario of the COVID-19 pandemic.

### Visits and diagnosis

The COVID-19 pandemic situation is exceptional and generates enormous pressure on health systems. A drastic change from face-to-face visits to telemedicine occurred in urology as well as in other specialties( 6 , 7 ). Depending on local regulations, telemedicine is a feasible option to triage patients, offering the advantages of reducing unnecessary hospital physical visits and the risk of transmission( 3 , 8 ). Counseling for asymptomatic patients, preoperative or post-operative guidance can be performed by teleconference. Medical expulsive therapy (MET) may be offered to patients with uncomplicated renal colic due to ureteral lithiasis. However, obviously a main limitation of teleconferences is to conduct a properly physical examination.

Ancillary test, like ultrasound, X-ray, lab analysis or CT scan could be addressed, but may be limited and should be requested in a rationale way under the current situation of the outbreak. Face-to-face visits are expected to increase again as the incidence drops and patients feel safe. However, telemedicine may continue to be implemented after the outbreak.

Keep patients and urology team safe. For face-to-face, it is not necessary to test all patients attending, but it is highly recommended that suspicious symptoms (fever, cough, headache, muscle pain, diarrhea, conjunctivitis, anosmia, or loss of taste) and contact with COVID-19 patients should be asked by phone before ( 3 ). Apply measures including a physical distance of 2 meters, frequent hands washing, longer time between patients and limit waiting room occupancy is recommended. Patients should wear mask and gloves and health professionals properly personal protective equipment (PPE) based on recommendations ( Table-1 ), including masks (preferably FPP3), shield and gloves ( 3 ).

**Table 1:** Personal Protective Equipment (PPE) recommended for health professionals, in contact with patients during covid-19 outbreak.

	Front office staff working	At face to face visit	Performing endoscopic procedures	Anestesiologist performing intubation	Assigned to take biological samples from COVID-19 patient or laboratory personnel in contact with samples	In contact with suspected or confirmed case of COVID-19	In contact with symptomatic patient (cold, cough, fever)
Physical distance of > 1.5 mt if possible	+++	+++	-	-	-	+++	+++
Patient should wear a mask / provide a mask to the patient	+++	+++	+++	+++	+++	+++	+++
Wear ffp2 mask	+++	+++	+++	+++	+++	+++	+++
Wear ffp3 mask	+	++	+++	+++	+++	+++	+++
Frequent hand hygiene, washing or by using 60% alcohol solution	+++	+++	+++	+++	+++	+++	+++
Wear Gloves	+++	+++	+++	+++	+++	+++	+++
Wear Double gloves	+	+	++	++	+++	+++	+++
Wear goggles, shield or barrier to protect eyes from biological liquids splashes	++	++	+++	+++	+++	+++	+++
Water repellent coat	+	++	+++	+++	+++	+++	+++

highly recommended/mandatory = +++, medium recommended = ++, weak recommended/optional = +

### Indications and prioritization of surgeries

With the determination to postpone all elective surgical procedures in most affected countries by the COVID-19 pandemic, the term “elective” is open to different interpretations. Elective procedures can be stratified as “essential”, which implies that there is an increased risk of adverse outcomes when delaying surgical care for an indefinite period of time, compared to “non-essential”, which alludes to purely elective procedures that are not time sensitive for medical reasons ( 9 ).

In this scenario, urologists around the World will have to choose which surgeries should be maintained in the current circumstances ( 2 ). It is necessary to discuss the topic in order to minimize the impact and risks for patients and health professionals who provide urological care. Indications and prioritization of lithiasis surgery during COVID-19 outbreak are summarized in Table-2 .

**Table 2:** Indications and Prioritization of lithiasis surgery during COVID-19 outbreak.

TREATMENT				
Priority Category	Low	Intermmediate	High	Emergency
	Up to 6 months	4 to 12 weeks	2 to 4 weeks	Up to 24 hours
COVID - RECOMMENDATIONS				
**Drainage- JJ Catheter or Nephrostomy**				Obstructive ureteral stone with infection Sepsis due to obstructive stone, anuria Post-operative complications (abscess, fistula, avulsion)
**Urgent descompression (JJ Catheter or Nephrostomy) or endourological stone removal**			Obstructive ureteral stone failed MET (> 4 weeks) or too large to pass (e.g. > 08-10mm) Symptomatic ureteral stone, not controlled with medication, or recorrent ED visits Obstructive ureteral stone with AKI Recorrent infections in obstructive ureteral stone despite drainage and antibiotics Staghorn stones with uncontrolled infection Patients with nephrostomy (obstructive stone) or PCNL 2nd time	Obstructive ureteral or pyelic stone in solitary kidney, bilateral ureteral obstruction and intractabal symptoms requiring admission
**Treatment - endourological stone removal**		Ureteral stone, symptoms controlled, undergoing trial of MET Ureteral stone with pre-existing stent with stent associated symptoms requiring medications Recurent infections in non obstructive renal stones requiring antibiotics and with worsen renal function Renal stones causing intermitent obstruction		
**MET**		Asymptomatic non-obstructive ureteral stone		
**Interventional treatment (SWL, URS F-URS and PCNL)**	Asymptomatic / oligoasymptomatic renal stones (without UTI and worsen of the renal function) Majority of the stones requiring PCNL and F-URS			
**JJ Catheter removal (endourological intervention when necessary)**	Patients with JJ catheter with periodic changes	Patients with JJ catheter requiring definitive treatment Ureteral or renal stone with pre-existing stent with well tolerated symptoms		Patients with JJ catheter requiring hospitalization (pain, infection and severe haemathuria)

**Observations:** Stone treatment is preferred over drainage to diminish the ED visits SWL has lower stone free rate and higher rate of secundary stone treatment, so URS is preferred Always consider the risk group in order to indicate the surgery (immunocompromise, diabetes, renal dysfunction) Consider stenless or stent-on-string to avoid clinic visit when possible

According to the American College of Surgeons ( 10 ) to assess the real impact of the pandemic on elective procedures, it is important to understand the availability of resources at local facilities, such as: number of beds, tests, operating rooms, as well as their restrictions, such as work-force, supply chain, etc. In addition, the cancellation of surgeries contributes to social isolation and saves resources such as personal protective equipment (PPE) for the care of patients with COVID-19 infection.

Numerous patients with confirmed or suspected COVID-19 will require urgent surgical treatment ( 11 ). All postponable procedures must be rescheduled in order to reduce the exposure of the surgical team and the patient to potential contamination ( 4 ). A delay in procedures after the end of the COVID-19 pandemic is inevitable, and hospitals must plan how to deal with it effectively, ensuring that patients who has elective treatment presents the best possible results ( 12 ). Undoubtedly, the consequences of reckless elective surgery cancellations can have a more dramatic and immeasurable impact on the health of our communities than the morbidity and mortality inflicted by the new disease caused by the new coronavirus ( 9 ).

Some actions must be taken in order to reduce the spread of the disease during surgery, such as: using as much disposable material as possible, keeping the operating room doors closed, except for the circulation of employees, patients and instruments. Never handle instruments without gloves, avoid using cutting materials when possible ( 11 ). To define the indications for endourological procedures, it is necessary to proceed with the same care. Published studies on the detection of SARS-CoV-2 in urine are inconclusive and the evidences are not yet robust. Only one Chinese study reported the presence of viral RNA in urine samples of approximately 6.9% of infected patients ( 13 ).

In the last decades, elective and emergency admissions related to urolithiasis have increased ( 14 ). Uro-sepsis due to untreated obstructive pyelonephritis or a calculus matrix with bacterial colonization are more frequent than in the past ( 15 ). Urolithiasis patients scheduled for surgery should be carefully selected according to surgical priority. Although urinary lithiasic disease represents a benign condition, in a significant number of cases it can lead to potential serious septic complications that could increase the burden on emergency services ( 1 ). Therefore, it is recommended that endoscopic procedures and urethral catheterization be performed with caution and surgeons be fully protected against infections if the patient is suspected or confirmed COVID-19 ( 3 ).

Fever can be a confusing factor in the surgical indication of patients with urinary tract obstruction, since this symptom may be due to COVID-19 and not due to bacterial infection ( 16 ). It is worth mentioning that, even with the decompression of the urinary system, antibiotic therapy and other supportive actions, 15% of these patients with sepsis require admission to the intensive care unities (ICU), with a mortality rate of 8 to 10% ( 15 ).

Therefore, most urolithiasis surgical treatments should be suspended, unless they are emergency surgeries, as in the case of obstructive pyelonephritis. In this case, one must choose to drain the urinary tract with a double J catheter implant under spinal or even local anesthetic block. Percutaneous nephrostomy can be considered when indicated (local anesthesia). Cases of ureteral obstruction in a single kidney, bilateral ureteral obstruction, acute impairment of renal function and refractory pain to clinical treatment should not be delayed. The remainder of cases of acute flank pain should preferably be treated clinically with medical expulsive therapy (MET) ( 4 ).

In situations when ureteral catheters are needed, the use of double J with external wire should be considered, to reduce the need for an additional procedure to remove it. In many cases, the patient would be able to remove the stent at home, avoiding a new visit to the hospital ( 13 ). Patients with indwelling catheter prior to the COVID era, without symptoms or oligosymptomatic, may stay with the catheter longer if necessary; on the other hand, for cases operated during the pandemic, we should try to remove a double J catheter as soon as possible, on an outpatient basis with local anesthesia, without the need for hospitalization ( 17 ).

The ideal moment to return to elective surgical activities is still uncertain. Many studies about this subject have been elaborated with many researchers and authorities involved ( 18 ). Recently, a study published in The Lancet on the detection of SARS Cov-2 viral RNA in patients healed from the disease, who had moderate (n = 46) or severe (n = 30) symptoms, demonstrated that neither group presented the viral material after the 25th day of symptom onset and that 90% of patients with mild condition tested negative for Covid-19 on the tenth day of symptom onset ( 19 ).

We believe that as there is no significant scientific evidence of viral elimination in the urine and based on the reported data on the criteria for curing the disease, elective endourological surgery in urinary lithiasis could be safely performed after 30 days at the onset of symptoms, however, we suggest that new randomized studies are taken as a reference for the topic ( 19 ). Some strategic changes to contain the spread of SARS Cov-2 can be consolidated as permanent, including in the structure of care of the operating rooms, use of computerized tools for virtual follow-up in the postoperative period and the managerial awareness of hospital resources ( 20 ).

### Strategy for pre-surgical screening during Covid-19 pandemic

**What should be the ideal screening of patients who undergo surgery during COVID-19 pandemic?.**

Ideally, each patient should receive a telephone triage in which symptoms suggestive of COVID-19 are investigated such as cough, fever, shortness of breath, diarrhea, conjunctivitis. Telephone triage should also investigate contact with positive or at risk COVID 19 patients. Once ruled out by telephone any symptom at the time of admission, a triage should be repeated, evaluating the possible onset of symptoms from the phone call to admission.

Considering that a large portion of the population could be COVID-19 positive but still asymptomatic, ideally each patient should enter the hospital in droplet isolation, receive two nasopharyngeal swabs and be considered positive until they have negative double swab results. Although behaving in this way, two nasopharyngeal swabs have a diagnostic accuracy of about 65%, it means that 35% of asymptomatic positive patients would not be identified. A model of this type is very difficult to realize as well as expensive and not 100% safe. Various strategies have been described so far in the literature( 1 , 16 , 21 ), but no one can be considered the gold standard. In real life, telephone triage is certainly a valuable and indispensable resource in identifying suspicious cases and should certainly be pursued.

Entrance to the hospital should be permitted by a single access and subject to strict controls. At the time of admission, a triage should be repeated. Body temperature should be checked for all patients. Every patient should wear surgical masks which should be provided by the hospital and have access to 60% alcohol solutions for hand hygiene. Personal Protective Equipment (PPE) and social distance are simple and indispensable measures to avoid the spreading of the infection. A task force dedicated to COVID-19 should be set up in each hospital in order to identify and manage any suspicious case.

Visitors should be allowed to access to the hospital after strict controls just for a limited span of time and only one person for patient. They should follow the same rules of admission valid for patients.

### Surgery preparation and course (Consider Covid19 negative and Covid19 patients)

First of all hospitals should be split in COVID free hospitals and COVID hospitals. COVID 19 patients should not undergo surgery except for emergencies not possible to postpone.

In case of an emergency any confirmed or suspected COVID-19 patient requiring urgent endourological surgery should be treated in a dedicated operative room (OR) with a negative pressure environment and separate access from the other ORs. For hospitals in which a dedicated OR is not available, patients should be transferred or otherwise, if not possible, all postoperative cleaning protocols should adhere to institutional central disease control instructions.

All surgeries should be performed preferably from the best surgeon available, not in learning curve, to shorten operative time and reduce complications ( 1 , 22 ).

In COVID free hospitals we suggest to use the following PPE from different HWs ( Table-1 ). The anesthesiology protocols should limit aerosolization as much as possible. PPE that anesthesiologists should use during intubation are described in Table 1. Airway management should follow strict rules leading to achieve RSI (rapid sequence intubation) to reduce aerosolization.

Extubating should be performed in OR. (High efficiency particulate air filters) HEPA should be applied on each oxygen system interface (circuit, mechanical fan) and changed for every patient. Despite overall benefit of high-flow nasal oxygen, a nasal oxygen at 3 l/min should be preferred to avoid high flow aerosol-generating technique. A disposable video laryngoscope with a separate screen should be used to minimize patient contact( 23 ).

The sterilization of the surgical material should not be different from that usually performed even if the use of disposable instruments and equipment would be preferable. Evacuation of irrigation fluid during endourological procedures should be collected through a closed system ( 3 ).

During the outbreak of the pandemic scenario elective stone procedures such as RIRS and PCNL should be postponed. As regards the treatment of obstructive ureteral stones, ureteral stents and nephrostomy tubes insertion should be preferred to uretherolithotripsy. Not knowing how long the health emergency will last, it would be ideal to place long-lasting stents or nephrostomies so as to reduce the risk of incrustations or malfunctions of the same.

It is likely that postponing all elective interventions for stone disease will lead us to face more complex cases later and that the waiting lists become even longer than they already were. Presence of virus in urine is controversial. Viral load seems to be low but present in urine( 24 , 25 )

### Residents role

Activity reduction or suppression of outpatients and elective surgeries brought to a generalized slowdown of residents'activity ( 26 ). Surgical and academic activity of residents before the pandemic were just not ideal in most countries ( 27 - 31 ).

Amparore et al. found that Italian residents experienced a severe reduction or complete suppression of training exposure for clinical and surgical activities because of COVID pandemic ( 32 ).

In this scenario surgeries should be performed from the best surgeon available to shorten operative times and reduce the complications ‘risk, a lack of a structured mentorship was just previously associated with an increased risk of residents ‘burn out and will potentially expose residents to higher burn out risk ( 33 ).

Suggestions have been made to implement and integrate residents' activity with web platforms made available from EAU and ESU (European School of Urology) and through the use of Social Media as scientific platform ( 34 ). We believe residents in this scenario should carry on to shadow their mentors and try to get all the possible teachings from an extraordinary situation.

## FOLLOW-UP OF PATIENTS

### Asymptomatic patients in follow-up

Medical practices have been largely affected since the beginning of the Covid-19 pandemic, with the postponement or cancellation of medical visits. Centers severely affected by the pandemic offer consultations for follow-up of malignant diseases, and screening is carried out before the visit, so that only patients without fever or respiratory symptoms can be attended in face-to-face visits. All consultations are individual, with doctors wearing PPE. In some situations, telephone consultations and videoconferences can be used ( 16 , 35 ).

Telephone consultations were also used to screen the urgency of surgical treatment. To avoid further hospital visits, imaging tests performed before the pandemic were used in conjunction with telephone consultations and routine follow--up were postponed ( 36 ).

### Schedule for the follow-up visit

During the pandemic, clinics maintain daily waiting lists for patients to be treated according to internal prioritization guidelines. This procedure allows dynamically and together with other disciplines to react to changes in operational capabilities and to treat patients within the appropriate time frame. Other individual aspects must be taken into account by the physicians in charge. This includes parameters related to the patient, such as age, previous illnesses or individual opinions of the patient, as well as the consideration and availability of alternative non-operative therapies (for example, active monitoring, radiotherapy), drug therapy or neoadjuvant approaches. In addition to the transfer of urological patients to other facilities, the exchange of employees must also be considered, as it is already being prepared in many places in Germany ( 37 ).

In countries affected by the pandemic, such as Italy, hospitals were almost entirely dedicated to the treatment of patients with COVID-19, so that only emergency services were available for operated patients. It became necessary to remove urological patients across the country for emergency therapy ( 16 ). Relocating urological patients is generally much easier than removing patients with COVID-19. However, it is important to be aware of the patient's duty of care and solidarity at all times ( 37 ).

Adequate urological care should be provided to the patient, despite the fear that some have of infection or of finding an overburdened health system. Urologists need to use their communication channels to advise patients to only go to hospitals with acute complaints or in urgent cases. The dissemination of information through homepages, online portals and newsletters is an adequate action to inform patients about the availability of urological care ( 37 ). In times of general uncertainty, the appointments over the phone or online can be used as a substitute for face-to-face visits. The objective is to consider alternatives with the respective patient and define a common strategy in the current situation.

Ambulatory consultations by electronic means are inevitable in the current situation. Due to general uncertainty, many patients cancel non--urgent appointments to reduce their own risk of infection when they contact health care providers. Most patients show an understanding of the current health system situation. The inclusion in waiting lists or the allocation of future consultations can serve as an instrument for patient adequacy and safety. In many clinics, consultations for patients with tumors are now carried out exclusively by electronic means ( 37 ).

### Cystoscopy and stent removal schedule

Patients who already had a ureteral stent due to complicated urolithiasis before the COVID-19 pandemic, can lead to significant morbidity, such as acute pyelonephritis, bacteremia, urosepsis and even death ( 38 ). Therefore, this subset of patients should be considered with some priority, in order to avoid a prolonged delay. The length of time the stent remains should be a factor in the prioritization process, keeping in mind that most ureteral stents can be left in place for up to 6 to 12 months. Currently, although the evidence is insufficient to support antibiotic prophylaxis in patients with long-term stents, due to the likely delays in surgery, it can be considered to reduce the risk of urosepsis and the consequent need for a mechanical ventilator ( 1 ).

The stent with external wire must be considered after procedures without complications (stone-free) to avoid a visit to the clinic for its removal. Therefore, endourologists need to be prepared to subsequently manage more difficult cases for patients whose procedure has been postponed due to lower surgical priority; in addition, if the waiting list becomes large, one can try to anticipate the procedure. However, these patients should be routinely followed up by phone calls to monitor their status ( 1 ).

Standard sterilization of the reusable endourological arsenal is also considered safe in terms of cross-contamination with COVID19, because so far the virus has not been detected in urine, although the evidence is not yet robust ( 39 ).

### Follow-up tests: time in the context of the pandemic and when the outbreak goes down

Approximately 80% of patients with Covid-19 have mild disease, although the elderly and patients with comorbidities are at high risk of deterioration ( 40 ). The WHO (World Health Organization) clinical classification for the disease includes: mild disease, pneumonia and severe pneumonia, which is further categorized in adults and children ( 41 ). As most cases have mild symptoms, a high index of suspicion is required and all patients with fever and / or respiratory symptoms should be treated as having COVID-19 until proven otherwise ( 42 ). The most common symptoms include fever, cough, dyspnea, myalgia and fatigue ( 43 ). Gastrointestinal symptoms are not common, however, patients may experience nausea or diarrhea one to two days before the onset of fever and acute respiratory disease ( 42 ).

Laboratory diagnosis is necessary to confirm the diagnosis of COVID-19. The polymerase chain reaction with real-time reverse transcription (RT-PCR) is used to analyze nasopharyngeal or oropharyngeal aspirates in outpatients. In severe cases, lower respiratory samples of sputum and / or endotracheal aspirate or bronchoalveolar lavage may be used. If a patient with a high level of suspicion of COVID-19 has a negative result, additional samples should be sent (such as blood, feces and urine). To exclude COVID-19, the guidelines recommend two consecutive negative tests, which are performed at least one day apart ( 42 , 43 ).

All patients prioritized for surgical procedures should be tested with nasopharyngeal aspiration for COVID-19, if possible. The maximum capacity of urological hospital beds should be reorganized to reduce the number of beds for an adequate social distance between patients ( 16 ).

The types of tests we have should take into account several parameters, such as whether the test detects the infection directly (like the virus itself) or indirectly (like host antibodies), the test response time, the ability to run multiple tests at the same time (i.e. productivity), the need to have a minimum number of samples before testing (i.e. in batches) and the ability to perform the test in environments ( 44 ).

In order for the test results to allow for a specific clinical decision, researchers, the development of epidemiological policies and physicians need to consider each one with respect to the intention to test and the population being tested in the most specific way possible. At the moment, the detection of host-derived antibodies directed against SARS-CoV-2 will be crucial for surveillance, epidemic prediction and determination of Immunity ( 45 ).

## CONCLUSION

Categorization and prioritization of patients affected by lithiasis is crucial for management, surgical selection and follow-up. Protocols, measures and additional efforts should be carried out in the current situation of the COVID-19 pandemic.

## Abbreviations

RIRS: Retrograde Intra-renal Surgery
PCNL: Percutaneous nephrolithotomy
OR: Operating Room
PPE: Personal Protective Equipment
SARS-CoV-2: Severe Acute Respiratory Syndrome–Related Coronavirus-2
